# Development and external validation of a machine learning model for three-tier disability risk stratification in older adults

**DOI:** 10.3389/fpubh.2026.1904998

**Published:** 2026-09-18

**Authors:** Yitong Mao, Zhiting Guo, Jiayi Wang, Li Yang, Jiayi Chen, Yiming Lu, Yuping Zhang, Jingfen Jin

**Affiliations:** 1 Nursing Department, The Second Affiliated Hospital of Zhejiang University School of Medicine (SAHZU), Hangzhou, Zhejiang, China; 2 Key Laboratory of the Diagnosis and Treatment of Severe Trauma and Burn of Zhejiang Province, Hangzhou, Zhejiang, China; 3 Zhejiang Provincial Clinical Research Center for Emergency and Critical Disease, Hangzhou, Zhejiang, China

**Keywords:** disability, machine learning, older adults, risk prediction, risk stratification

## Abstract

**Background:**

Global aging has made disability among older adults a major public health challenge. Existing prediction models often lack external validation and actionable risk stratification. This study aims to develop and externally validate an explainable, three-tier machine learning framework for disability risk prediction and stratification among older adults.

**Methods:**

The training set and internal validation set were derived from the longitudinal development cohort of the China Health and Retirement Longitudinal Study (CHARLS, *n* = 3,599). An independent cross-sectional survey (*n* = 2,066) was used for external validation of the model. Individuals 65 years of age and older were included. Disability was defined by basic activities of daily living (BADL). After feature selection, five candidate machine learning techniques were evaluated to identify the optimal model. The SHapley Additive exPlanations (SHAP) was used to interpret the optimal model. Ultimately, a three-tier risk stratification method was developed and verified using Decision Curve Analysis (DCA) and the Cochran-Armitage trend test.

**Results:**

LASSO regression identified seven critical predictors. The Extreme Gradient Boosting (XGBoost) achieved the optimal balance, achieving the optimal balance of predictive performance in the internal testing set (AUC = 0.737) and external validation cohort (AUC = 0.782). SHAP analysis revealed age, age-adjusted Charlson Comorbidity Index (ACCI), and lower limb mobility as the paramount global predictors. The established risk stratification thresholds (33.17 and 65.23%) successfully categorized older adults into three levels. This three-tier system exhibited a significant gradient increase in actual disability incidence within the external set (*P*_trend_ < 0.001) and a broad positive net clinical benefit in DCA.

**Discussion:**

The XGBoost model showed satisfactory and consistent performance in external validation. SHAP provided clinically meaningful insights, emphasizing age, lower-limb mobility, psychological status, and comorbidity burden. The validated three-tier system bridges the translational gap between complex predicted probabilities and practical clinical decisions.

## 1 Introduction

With the accelerating global aging trend, disability among older adults has emerged as a critical public health challenge (1). Disability is generally defined as limitations in performing basic activities of daily living (BADL) and/or instrumental activities of daily living (IADL) due to physiological, psychological, or social factors (2, 3). With advancing age, individuals commonly experience a decline in health or a gradual loss of the ability to perform basic daily activities, such as bathing or doing housework (4). According to the Chinese Center for Disease Control and Prevention, the number of older adults with disabilities reached 52.71 million in 2020, and this figure is expected to exceed 77.65 million, accounting for 57% of the total disabled population by 2030 (5). Beyond its impact on individual independence, disability is closely associated with adverse outcomes such as depression, reduced quality of life, increased hospitalization rates, and elevated mortality risks (6, 7). Notably, disability during aging is not an isolated event but a dynamic process resulting from the long-term accumulation of multiple risk factors and complex nonlinear effects (8). Consequently, the accurate identification of disability risk and the development of targeted early-stage interventions are crucial for healthy aging.

In recent years, some researchers have explored and established risk prediction models for disability among older adults. Statistical models such as traditional logistic regression (9, 10) and Cox proportional hazards regression (11) are widely used to develop disability prediction tools. However, these traditional methods often struggle to capture high-dimensional variables and the complex nonlinear interactions among multiple risk factors (12). Although machine learning (ML) algorithms have been increasingly applied to improve predictive performance, several key limitations remain in current research. First, insufficient model generalization and a lack of external validation are the most typical issues. Many models lack independent external validation across cohorts with differing baseline characteristics, which makes it challenging to enable their applicability across regions and people (13–16). Second, in terms of modeling techniques, the majority of research rely on a single pre-specified algorithm without model selection, which may introduce selection bias (15, 17). Besides, the majority of binary predictive models now fail to convert these probabilities into clinical judgements that can be put into practice, i.e., to develop thresholds for risk stratification. This makes it more difficult to integrate predictive models with practical graded intervention strategies, which call for a significant differentiation between groups at low, moderate, and high risk.

To address these limitations, this study aims to develop and externally validate an explainable multi-model machine learning framework designed for the precision prediction and robust stratification of disability risk among older adults. Using representative longitudinal cohort data, we systematically evaluated multiple candidate algorithms to identify the optimal model. Based on the best-performing model, we applied the principle of maximum differential to establish thresholds designed to translate predictive probabilities into a practical three-tiered classification system (low, moderate, and high risk). Additionally, we integrated the SHapley Additive Explanation (SHAP) method to elucidate the relative contributions of risk factors at both the global and individual levels. Finally, to ensure the generalisability and transferability of our findings, this risk stratification framework will undergo rigorous validation in an independent external cohort.

## 2 Methods

### 2.1 Study design

This study followed a two-phase hybrid design comprising model development using a prospective longitudinal cohort and external validation using an independent retrospective cohort study. The study was conducted and reported in accordance with the Transparent Reporting of a multivariable prediction model for Individual Prognosis or Diagnosis (TRIPOD) statement (18) (Supplementary Appendix 1).

### 2.2 Participants and eligibility criteria

#### 2.2.1 Development and internal validation dataset

Participants for model development and internal validation were selected from the nationally representative longitudinal cohort–the China Health and Retirement Longitudinal Study (CHARLS) (19). CHARLS started in 2011 and used a multistage, probability-proportional-to-size (PPS) sampling technique with 17,708 participants who were 45 years of age or older from 28 provinces in China (19). Follow-up surveys are conducted every two to three years as part of the ongoing CHARLS investigation, which was updated in 2013, 2015, 2018, and 2020. The CHARLS data were collected by trained interviewers through face-to-face household interviews using a structured questionnaire and Computer-Assisted Personal Interviewing (CAPI) technology. This study included the 2015–2018 longitudinal waves of the CHARLS database. Participation in both the 2015 baseline survey and the 2018 follow-up, as well as being 65 years of age or older at baseline, were the requirements for inclusion. This age threshold was selected to align with the official aging statistics of Zhejiang Province (20), ensuring our findings are directly comparable with the regional demographic context. Individuals who were already classified as disabled at the 2015 baseline were excluded. Additionally, those who had incomplete information about the outcome indicators (disability status) during the 2018 follow-up were not included. Based on these standards, a final longitudinal cohort of 3,599 eligible older adults was included.

#### 2.2.2 External validation dataset

For the external validation dataset, participants were consecutively recruited from five community healthcare centers in Hangzhou and Huzhou City, Zhejiang Province, between July 2024 and December 2025. Age (65 years and older) and the absence of disability at baseline (based on a 1-year retrospective assessment) were the same inclusion criteria as those for the development cohort. Participants were excluded if they had severe cognitive or communication impairments that prevented them from accurately recalling their health status from the previous year. Baseline predictor variables were obtained by combining participants' own recollections of conditions from one year prior with records from the community health care system. Current disability status was assessed as an outcome at the time of the survey. This design maintains a clear temporal sequence (baseline predictors one year ago → outcomes now), and its one-year observation window roughly aligns with the forecast horizon expected by the model. Ultimately, a total of 2,066 participants met the eligibility criteria and were included in the external validation dataset.

Data collection was carried out by our research team, comprising trained nurses and research assistants, who conducted face-to-face interviews with participants. All interviewers completed a two-day training led by the principal investigator, covering study objectives and questionnaire administration. For the historical data, we worked with community healthcare center staff who assisted in retrieving and abstracting relevant information from the electronic health records system; they received a brief orientation on the study's data requirements and the standardized abstraction format. Quality was maintained through weekly team meetings to address any ambiguities in questionnaire responses or record abstraction.

#### 2.2.3 Sample size

Adequacy was confirmed using the events-per-variable (EPV) criterion: the development cohort provided 743 disability events (20.6%) against 46 candidate predictors, giving 16 EPV overall (11 in the training set) and exceeding the conventional minimum of 10 EPV; after LASSO retained 7 predictors, EPV rose to approximately 106 (full cohort) and 73 (training set). The external validation cohort (*n* = 2,066) contained 205 events and 1,861 non-events, satisfying the recommended threshold of at least 100 events and 100 non-events.

### 2.3 Outcome measure

The BADL measures the ability of the respondent to carry out everyday activities, such as dressing, bathing, eating, getting out of bed, using the restroom, and controlling urination and feces. Each task's replies from the participants were divided into four categories: (1) “No, I don't struggle.” (2) “I struggle, but I can still succeed.” (3) “Yes, I need some assistance” and (4) “I am unable to do it.” Any responder who scored a 2, 3, or 4 on any BADL item was deemed to have a disability (21).

### 2.4 Potential predictors

Based on a comprehensive literature review of risk factors for disability (11, 16, 22, 23) and data availability in the CHARLS database, a total of 46 candidate predictor variables were initially extracted for feature selection. These variables were categorized into the following six domains: (1) sociodemographic characteristics (*n* = 6): age, gender, marital status, education level, retirement status, and history of accidents; (2) lifestyle factors (*n* = 4): smoking status, drinking status, and participation in social activities; (3) family and economic support (*n* = 4): family member number, co-residence with children, having children living nearby, and financial support; (4) comorbidities and hospitalization (*n* = 12): history of 10 specific chronic conditions (hypertension, stroke, diabetes, chronic lung diseases, cancer, psychiatric illnesses, heart disease, digestive diseases, arthritis, and memory-related diseases). Additionally, the overall comorbidity burden was quantified using the Age-adjusted Charlson Comorbidity Index (ACCI) (24, 25), and hospitalization history was also included; (5) health status and functional limitations (*n* = 12): self-reported physical limitations, including difficulty in extending arms, lifting weights, picking up a small coin, and overall upper mobility, self-reported orientation, and depression; (6) physical examinations (*n* = 8): body mass index (BMI), waist circumference, abdominal obesity, and balance test.

The measurement of some of the predictor variables is as follows. Participation in social activities was assessed by asking, “Did you participate in any of the following activities last month?” Examples include socializing with friends, playing cards or board games, joining community clubs, doing volunteer work, and attending educational or training courses. Orientation was assessed by asking participants about their current year, month, day, day of the week, and season. Getting all the answers correct is considered no problem. The 10-item Center for Epidemiologic Studies Depression (CESD-10) Scale (26) was used to measure depressive symptoms. Participants were asked to rate the frequency of each symptom on a four-point scale. It is advised that the criterion for diagnosing depressed symptoms be set at 10 or above. Based on performance, the balance test was divided into four levels of an ordinal score: (1) unable to finish the semi-tandem or side-by-side stand in the allotted time; (2) unable to finish the semi-tandem stand but able to finish the side-by-side stand; (3) able to finish the semi-tandem stand but not the full tandem stand; and (4) able to finish both the semi-tandem and full tandem stands in the allotted time. Better balance function is indicated by a higher score (27). All data in CHARLS were collected through face-to-face household interviews using a structured questionnaire and Computer Assisted Personal Interviewing (CAPI) technology. Data in the external validation dataset was collected through on-site, one-on-one interviews conducted by trained researchers. The evaluation criteria and guidelines for all predictor variables in the external validation dataset were consistent with those of the training set. To ensure transparency and reproducibility, a detailed codebook including the evaluation criteria, operational definitions, and scoring/encoding methods for all 46 variables is provided in Supplementary Appendix 2.

### 2.5 Statistics analysis

All data analyses were performed with R software, version 4.6.0 (R Foundation for Statistical Computing, Vienna, Austria). Continuous variables were presented as mean ± standard deviation (SD) for normally distributed data or as median with interquartile range (IQR) for non-normally distributed data. Categorical variables were expressed as frequencies and percentages. Analysis of Variance (ANOVA) and chi-square tests were used to compare the differences between groups for continuous variables and categorical variables, respectively. Missing data was handled using Multiple Imputation by Chained Equations (MICE) with the random forest method (method = “rf”). A total of five imputed datasets were generated (m = 5), with 10 iterations per dataset (maxit = 10). The imputation model included all candidate predictors and the outcome variable. Convergence was assessed by trace plots of the mean and variance across iterations. A sensitivity analysis was conducted by varying the parameters of the interpolation model. Continuous variables underwent Z-score standardization to eliminate scale-dependent biases. To minimize the impact of multicollinearity on the interpretability of the model, a Pearson correlation analysis was conducted prior to feature selection, and redundant variables with correlation coefficients greater than 0.9 were removed. A two-sided *P*-value < 0.05 was considered statistically significant.

#### 2.5.1 Model construction and evaluation

The processed CHARLS cohort was randomly partitioned into a training set and an internal testing set at a 7:3 ratio. A balanced class distribution was achieved using Synthetic Minority Oversampling (SMOTE). The steps for model building and evaluation were as follows: (1) The Least Absolute Shrinkage and Selection Operator (LASSO) regression was used for feature selection on the imputed training dataset in order to maximize model performance, reduce multicollinearity, and avoid overfitting (28). LASSO applies an L1 penalty to shrink the coefficients of less contributive variables to exactly zero. We employed a 10-fold cross-validation strategy to determine the optimal penalty parameter (λ) that minimized the mean squared error. (2) Five distinct machine learning techniques, including logistic regression (LR), random forest (RF), k-nearest neighbors (KNN), support vector machine (SVM), and extreme gradient boosting (XGBoost), were chosen to construct models. The optimisation of models was performed using 10-fold cross-validation with five repetitions and grid searching to adjust hyperparameters, ensuring stability. (3) The performance of the models was compared using multiple metrics: the Area Under the Receiver Operating Characteristic Curve (AUC), accuracy, sensitivity, specificity, F1-score, and Brier score (for calibration assessment). The Youden index was used to select the threshold, and the Delong test was used to compare the Receiver Operating Characteristic (ROC) curves of the training and testing sets to avoid overfitting.

#### 2.5.2 Model explainability

We used the SHapley Additive eXplanation (SHAP) approach to interpret the black box model with the greatest performance in order to better explain how each feature variable influences and contributes to the best model (29). Based on game theory, where each feature is seen as a participant, SHAP values calculate the impact of each feature on the prediction outcome. By equitably assigning prediction performance to each feature, SHAP explains how each feature contributes to a single observation. By calculating the mean absolute value of each feature's SHAP value, we were able to assess its significance. To gain a better understanding of the overall pattern and the range of features' impact on the dataset, we additionally displayed the SHAP values for each characteristic of each sample.

#### 2.5.3 Risk stratification

In order to convert continuous predicted probabilities into actionable clinical grades, the maximum selection rank statistic was set to (30), and a systematic grid search was conducted on the training set to determine the optimal threshold. Using a step size of 2%, candidate thresholds were assessed for every combination of the XGBoost predicted probability's 30th−60th percentiles (for the lower cut-off) and 60th−95th percentiles (for the upper cut-off). Participants were divided into three risk categories for each candidate threshold pair (t_1_, t_2_): Low-risk (< t_1_), Moderate-risk (t_1_ ≤ probability < t_2_), and High-risk (≥t_2_). This specific three-tier framework was chosen to balance statistical discrimination accuracy (31) with clinical practicality, avoiding the oversimplification of binary models while seamlessly aligning with standard three-level clinical care workflows. The actual disability rates for each of the three groups were then compared using a chi-square test. In order to ensure the maximum divergence in disability occurrence across the three risk tiers, the ideal threshold combination was chosen as the one that maximized the chi-square statistic.

The generalisability of this stratification was evaluated in the external validation cohort using the Cochran-Armitage trend test. Finally, Decision Curve Analysis (DCA) was performed to assess the clinical net benefit of the risk stratification framework across various threshold probabilities. The entire workflow of this study is displayed in Supplementary Appendix 3.

### 2.6 Ethical considerations

Participants in the CHARLS study gave permission for the secondary use of their data, and the study was approved by Beijing University's Biomedical Ethics Review Committee (IRB00001052-11015). The public can obtain CHARLS data by making a formal request at: http://charls.pku.edu.cn, the CHARLS home page. The collection of data for the external validation set has been approved by the Human Research Ethics Committee of the Second Affiliated Hospital of Zhejiang University School of Medicine (No. 20231268). Participants provided written informed consent, with the option to withdraw at any time. All data were anonymised and stored in password-protected files.

## 3 Results

### 3.1 Baseline characteristics

A total of 5,665 participants were ultimately included in the study, including 2,519 in the training set, 1,080 in the internal validation set, and 2,066 in the external validation set. The detailed sample selection process is shown in Supplementary Appendix 3. Detailed baseline demographic characteristics, lifestyle factors, comorbidities, and physical measurements for the three groups are presented in Table 1. The model development cohort's median age was 70.00 years (IQR: 67.00–74.00), with a slight male predominance (52.5%). The most common medical diseases were hypertension (40.9%) and arthritis (41.4%), combined with a noteworthy 31.4% incidence of self-reported depressive symptoms. The training and internal validation sets' distributions of all baseline characteristics were extremely consistent (*P* > 0.05), supporting the efficacy of the stratified random sampling and offering an objective basis for model training. In contrast, the external validation cohort exhibited significant covariate shifts and marked heterogeneity compared with the training set (*P* < 0.05). Participants in the external set were much older (median age 73.00 years) and had a greater percentage of females (52.9%) than those in the development cohort. Additionally, they reported significantly lower rates of depressive symptoms (3.0%), arthritis (2.5%), and combined lower and upper limb movement difficulties (16.9 vs. 55.4%) but a greater burden of metabolic chronic diseases, such as higher prevalences of diabetes (13.6 vs. 9.6%) and hypertension (57.6 vs. 40.9%). Among the 3,599 participants in the training and internal validation cohort, the proportion of missing data across all candidate predictors ranged from 0.0 to 15.3%. The variables with the highest missing proportions were household economic status (15.3%), waist circumference (12.7%), and BMI (9.7%). Detailed missingness patterns across all variables are presented in Supplementary Appendix 4.

**Table 1:** Baseline characteristics of the three datasets.

Variable	Level	Training (*N* = 2,519)	Internal validation (*N* = 1,080)	External validation (*N* = 2,066)	*P*_1_	*P*_2_
Age	/	70.00 [67.00, 74.00]	70.00 [67.00, 75.00]	73.00 [69.00, 78.00]	0.739	< 0.001
Gender	Male	1,323 (52.5)	572 (53.0)	973 (47.1)	0.836	< 0.001
	Female	1,196 (47.5)	508 (47.0)	1,093 (52.9)		
Marriage	Married	1,917 (76.1)	827 (76.6)	1,683 (81.5)	0.589	< 0.001
	Widowed	573 (22.7)	243 (22.5)	345 (16.7)		
	Divorced	8 ( 0.3)	5 (0.5)	20 (1.0)		
	Never married	21 (0.8)	5 (0.5)	18 (0.9)		
Education	Secondary education	2,321 (92.1)	997 (92.3)	1,682 (81.4)	0.783	< 0.001
	Upper secondary	140 (5.6)	62 (5.7)	354 (17.1)		
	Tertiary education	58 (2.3)	21 (1.9)	30 (1.5)		
Retirement	No	1,261 (50.1)	556 (51.5)	1,732 (83.8)	0.456	< 0.001
	Yes	1,258 (49.9)	524 (48.5)	334 (16.2)		
Accident	No	2,180 (86.5)	921 (85.3)	2,055 (99.5)	0.340	< 0.001
	Yes	339 (13.5)	159 (14.7)	11 (0.5)		
Smoking	No	1,815 (72.1)	772 (71.5)	1,994 (96.5)	0.758	< 0.001
	Yes	704 (27.9)	308 (28.5)	72 (3.5)		
Drinking	No	1,330 (52.8)	568 (52.6)	1,514 (73.3)	0.939	< 0.001
	Yes	1,189 (47.2)	512 (47.4)	552 (26.7)		
Drinking frequency	None	1,679 (66.7)	722 (66.9)	1,523 (73.7)	0.291	< 0.001
	Less than once a month	177 (7.0)	89 (8.2)	22 (1.1)		
	1–3 days a month	92 (3.7)	48 (4.4)	12 (0.6)		
	1–4 days a week	136 (5.4)	47 (4.4)	29 (1.4)		
	Equal to or more than 5 days a week	435 (17.3)	174 (16.1)	480 (23.2)		
Social activity	No	1,374 (54.5)	572 (53.0)	1,479 (71.6)	0.403	< 0.001
	Yes	1,145 (45.5)	508 (47.0)	587 (28.4)		
Family member number	/	2.00 [2.00, 3.00]	2.00 [2.00, 3.00]	2.00 [2.00, 4.00]	0.607	0.009
Living with child	No	1,343 (53.3)	572 (53.0)	1,125 (54.5)	0.875	0.460
	Yes	1,176 (46.7)	508 (47.0)	941 (45.5)		
Children living near	No	179 (7.1)	68 (6.3)	1,143 (55.3)	0.419	< 0.001
	Yes	2,340 (92.9)	1,012 (93.7)	923 (44.7)		
Economic support received	No	327 (13.0)	145 (13.4)	1,331 (64.4)	0.758	< 0.001
	Yes	2,192 (87.0)	935 (86.6)	735 (35.6)		
Hypertension	No	1,489 (59.1)	654 (60.6)	876 (42.4)	0.440	< 0.001
	Yes	1,030 (40.9)	426 (39.4)	1,190 (57.6)		
Stroke	No	2,428 (96.4)	1,038 (96.1)	1,965 (95.1)	0.759	0.038
	Yes	91 (3.6)	42 (3.9)	101 (4.9)		
Diabetes	No	2,277 (90.4)	976 (90.4)	1,786 (86.4)	1.000	< 0.001
	Yes	242 (9.6)	104 (9.6)	280 (13.6)		
Lung diseases	No	2,108 (83.7)	884 (81.9)	2,057 (99.6)	0.195	< 0.001
	Yes	411 (16.3)	196 (18.1)	9 (0.4)		
Cancer	No	2,484 (98.6)	1,066 (98.7)	2,015 (97.5)	0.949	0.010
	Yes	35 (1.4)	14 (1.3)	51 (2.5)		
Psychological diseases	No	2,471 (98.1)	1,061 (98.2)	2,059 (99.7)	0.871	< 0.001
	Yes	48 (1.9)	19 (1.8)	7 (0.3)		
Heart diseases	No	2,006 (79.6)	852 (78.9)	2,010 (97.3)	0.644	< 0.001
	Yes	513 (20.4)	228 (21.1)	56 (2.7)		
Digestive diseases	No	1,845 (73.2)	760 (70.4)	1,478 (71.5)	0.084	0.210
	Yes	674 (26.8)	320 (29.6)	588 (28.5)		
Arthritis	No	1,477 (58.6)	623 (57.7)	2,014 (97.5)	0.622	< 0.001
	Yes	1,042 (41.4)	457 (42.3)	52 (2.5)		
Memory diseases	No	2,435 (96.7)	1,054 (97.6)	2,001 (96.9)	0.169	0.784
	Yes	84 (3.3)	26 (2.4)	65 (3.1)		
ACCI	/	5.00 [4.00, 6.00]	5.00 [4.00, 6.00]	4.00 [1.00, 5.00]	0.050	< 0.001
Hospitalization	0	2,160 (85.7)	903 (83.6)	1,669 (80.8)	0.110	< 0.001
	≥1	359 (14.3)	177 (16.4)	397 (19.2)		
Walking difficulty	No	2,403 (95.4)	1,029 (95.3)	1,894 (91.7)	0.947	< 0.001
	Yes	116 (4.6)	51 ( 4.7)	172 (8.3)		
Stooping difficulty	No	1,827 (72.5)	764 (70.7)	1,838 (89.0)	0.292	< 0.001
	Yes	692 (27.5)	316 (29.3)	228 (11.0)		
Stair climbing difficulty	No	1,561 (62.0)	654 (60.6)	1,929 (93.4)	0.446	< 0.001
	Yes	958 (38.0)	426 (39.4)	137 (6.6)		
Chair standing up difficulty	No	1,942 (77.1)	828 (76.7)	2,002 (96.9)	0.814	< 0.001
	Yes	577 (22.9)	252 (23.3)	64 (3.1)		
Arm extension difficulty	No	2,341 (92.9)	995 (92.1)	2,038 (98.6)	0.436	< 0.001
	Yes	178 (7.1)	85 (7.9)	28 (1.4)		
Heavy lifting difficulty	No	2,227 (88.4)	939 (86.9)	1,901 (92.0)	0.238	< 0.001
	Yes	292 (11.6)	141 (13.1)	165 (8.0)		
Coin picking up difficulty	No	2,455 (97.5)	1,045 (96.8)	2,029 (98.2)	0.287	0.105
	Yes	64 (2.5)	35 (3.2)	37 (1.8)		
Number of difficulties in lower limb movement	0	1,197 (47.5)	499 (46.2)	1,743 (84.4)	0.491	< 0.001
	≥1	1,322 (52.5)	581 (53.8)	323 (15.6)		
Number of difficulties in upper limb movement	0	2,084 (82.7)	863 (79.9)	1,893 (91.6)	0.049	< 0.001
	≥1	435 (17.3)	217 (20.1)	173 (8.4)		
Number of difficulties in lower and upper limb movement	0	1,124 (44.6)	457 (42.3)	1,716 (83.1)	0.215	< 0.001
	≥1	1,395 (55.4)	623 (57.7)	350 (16.9)		
BMI	/	22.66 [20.18, 25.22]	22.70 [20.14, 25.22]	23.31 [21.23, 25.59]	0.775	< 0.001
BMI level	Underweight (< 18.5)	277 (11.0)	116 (10.7)	119 (5.8)	0.987	< 0.001
	Normal weight (18.5–23)	1,076 (42.7)	468 (43.3)	823 (40.1)		
	Overweight (23–25)	485 (19.3)	207 (19.2)	486 (23.7)		
	Obesity (25+)	681 (27.0)	289 (26.8)	622 (30.3)		
Waist	/	84.80 [76.70, 92.00]	84.50 [76.00, 92.00]	83.00 [77.00, 90.00]	0.633	< 0.001
Abdominal obesity	No	1,475 (58.6)	638 (59.1)	1,309 (63.8)	0.800	< 0.001
	Yes	1,044 (41.4)	442 (40.9)	743 (36.2)		
Side-by-side test	No	10 (0.4)	5 (0.5)	84 (4.1)	1.000	< 0.001
	Yes	2,509 (99.6)	1,075 (99.5)	1,982 (95.9)		
Semi-tandem test	No	47 (1.9)	19 (1.8)	139 (6.7)	0.934	< 0.001
	Yes	2,472 (98.1)	1,061 (98.2)	1,927 (93.3)		
Full-tandem test	No	581 (23.1)	244 (22.6)	198 (9.6)	0.791	< 0.001
	Yes	1,938 (76.9)	836 (77.4)	1,868 (90.4)		
Balance level	Level 1	9 (0.4)	5 (0.5)	84 (4.1)	0.896	< 0.001
	Level 2	38 (1.5)	14 (1.3)	56 (2.7)		
	Level 3	541 (21.5)	226 (20.9)	65 (3.1)		
	Level 4	1,931 (76.7)	835 (77.3)	1,861 (90.1)		
Orientation	No	1,003 (39.8)	431 (39.9)	2,003 (97.0)	0.989	< 0.001
	Yes	1,516 (60.2)	649 (60.1)	63 (3.0)		
Depression	No	1,728 (68.6)	722 (66.9)	2,005 (97.0)	0.322	< 0.001
	Yes	791 (31.4)	358 (33.1)	61 (3.0)		
Disability	/	511(20.3)	232(21.5)	205(9.9)	0.417	< 0.001

Data after multiple imputation are presented as median [IQR] for continuous variables or “n” (%) for categorical variables. *P*_1_ represents the *P*-value for the comparison between the training set and the internal validation set; *P*_2_ represents the *P*-value for the comparison between the training set and the external validation set. The proportions of missing values for each variable in the original data are provided in Supplementary Appendix 4.

### 3.2 Feature selection

A Pearson correlation heatmap was created to visualize the pairwise correlations and evaluate their linear dependencies, as shown in Supplementary Appendix 5. Four highly connected redundant variables were eliminated, leaving 42 variables for additional screening. Subsequently, the LASSO regression was applied to these variables. Figure 1A displays the LASSO coefficient profiles of the candidate features against the λ penalty parameter (logλ). As the penalisation increased, the coefficients of less informative variables were progressively shrunk to exactly zero, effectively eliminating noise and redundant dimensions. To determine the optimal regularization parameter, a 10-fold cross-validation strategy was conducted. As depicted in Figure 1B, the partial likelihood deviance was plotted against log λ. To enhance the parsimony of the model and facilitate its clinical implementation, we specifically selected the λ value that yielded a smaller number of predictors—the value corresponding to one standard error from the minimum deviance to determine the final feature set. Consequently, the LASSO regression algorithm strictly selected a robust subset of seven critical predictors with non-zero coefficients. These seven optimal features were subsequently utilized for the development of the machine learning models. Although ACCI is age-adjusted, the correlation between chronological age and ACCI was low (r = 0.31; see Supplementary Appendix 5), indicating no significant collinearity. Both variables were retained by LASSO with non-zero coefficients, supporting their independent contributions to the model.

**Figure 1:**
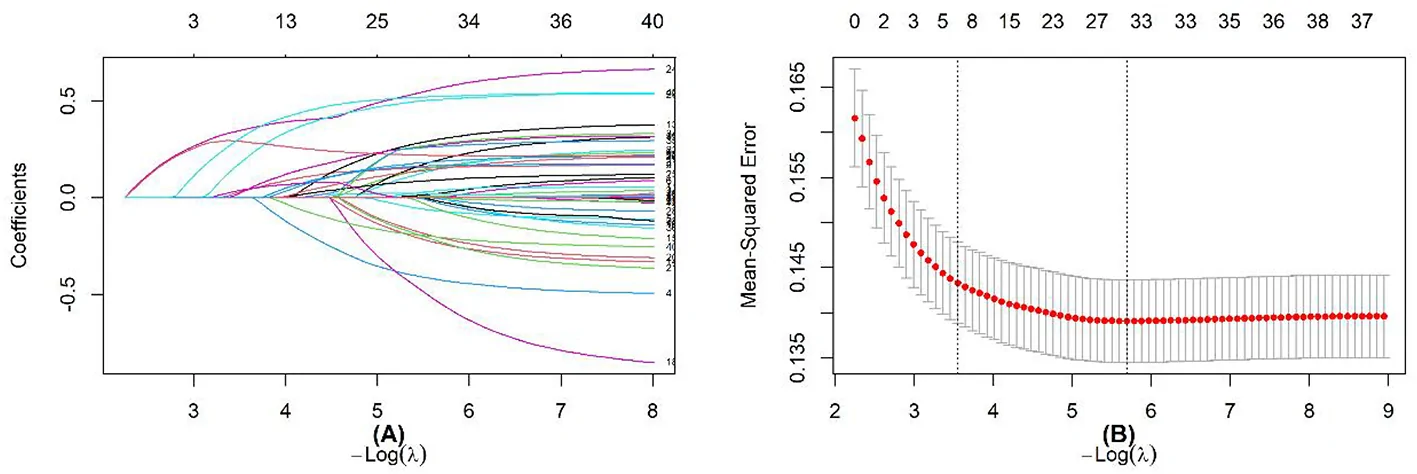
Variable selection by the LASSO regression model. **(A)** LASSO coefficient profiles of the candidate predictors; **(B)** Selection of the optimal λ by cross-validation.

### 3.3 Model evaluation

To identify the most robust predictive model, we conducted a comprehensive evaluation of the performance of five machine learning algorithms—LR, RF, SVM, KNN, and XGBoost. Detailed performance metrics, including AUC, accuracy, sensitivity, specificity, Brier score, and F1 score, are summarized in Table 2. The ROC curves for different prediction models are shown in Figure 2.

**Table 2:** Evaluation of the performance of the five algorithms.

Algorithm	Data set	AUC (95% *CI*)	Accuracy	Sensitivity	Specificity	Brier score	F1 score
LR	TS	0.754 (0.731–0.777)	0.689	0.693	0.688	0.223	0.475
LR	IS	0.734 (0.698–0.771)	0.674	0.690	0.670	0.198	0.476
LR	ES	0.786 (0.748–0.824)	0.794	0.673	0.808	0.143	0.394
RF	TS	0.826 (0.814–0.839)	0.734	0.725	0.743	0.177	0.732
RF	IS	0.706 (0.669–0.743)	0.701	0.552	0.742	0.200	0.442
RF	ES	0.712 (0.678–0.746)	0.728	0.286	0.918	0.184	0.337
SVM	TS	0.770 (0.675–0.784)	0.695	0.737	0.653	0.198	0.707
SVM	IS	0.711 (0.673–0.749)	0.636	0.750	0.605	0.218	0.470
SVM	ES	0.775 (0.734–0.817)	0.845	0.654	0.866	0.145	0.455
KNN	TS	0.695 (0.656–0.734)	0.712	0.585	0.748	0.212	0.463
KNN	IS	0.683 (0.644–0.723)	0.701	0.573	0.736	0.224	0.451
KNN	ES	0.701 (0.668–0.735)	0.631	0.693	0.624	0.243	0.271
XGBoost	TS	0.894 (0.885–0.904)	0.807	0.766	0.858	0.138	0.797
XGBoost	IS	0.737 (0.701–0.774)	0.716	0.660	0.731	0.146	0.499
XGBoost	ES	0.782 (0.743–0.820)	0.809	0.712	0.819	0.078	0.425

TS, training set; IS, internal testing set; ES, external testing set.

**Figure 2:**
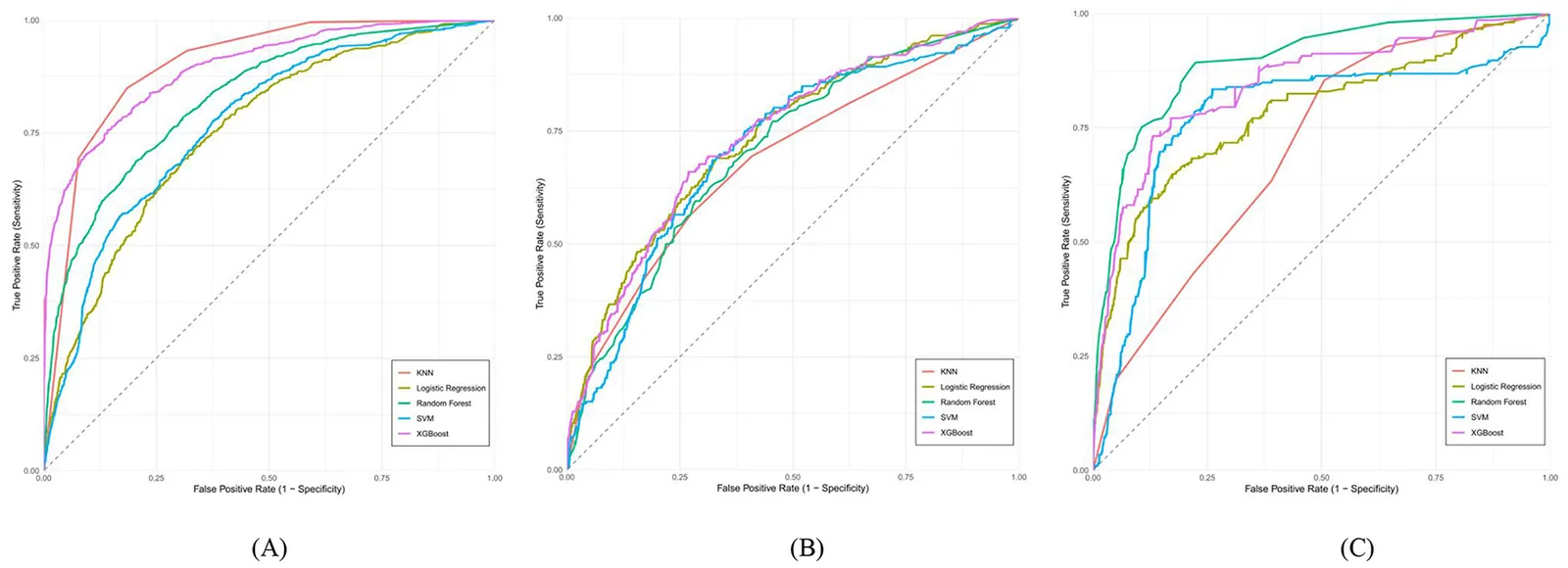
The ROC curves for different prediction models. **(A)** Training set; **(B)** internal testing set; **(C)** external testing set.

In the internal testing set, the XGBoost model demonstrated competitive predictive performance, achieving a comparable discriminatory power with an Area Under the Curve (AUC) of 0.737 (95% *CI*: 0.701–0.774) and a higher F1 score of 0.499. While the baseline LR model maintained stable performance (AUC: 0.734, F1: 0.476), other algorithms exhibited significant trade-offs. For instance, although the SVM model achieved the highest sensitivity (0.750) in internal testing, its specificity dropped to 0.605, indicating a higher propensity for false positives. In contrast, XGBoost provided a more balanced clinical profile, maintaining a sensitivity of 0.660 and a specificity of 0.731. The KNN model was the least effective, yielding the lowest AUC of 0.683 in the internal cohort.

The robustness of the models was further validated using an independent external cohort. XGBoost demonstrated satisfactory generalization and calibration, with an AUC of 0.782 (95% *CI*: 0.743–0.820) and a Brier score of 0.078, indicating high accuracy in probability estimation. Crucially, XGBoost maintained the highest sensitivity in the external cohort (0.712), outperforming the LR model (0.673) and the RF model, whose sensitivity dropped sharply to 0.286. These findings underscore the ability of the XGBoost algorithm to capture stable and highly discriminative clinical phenotypes across diverse populations. Consequently, the XGBoost model was chosen as the optimal framework for subsequent explainability analysis and the development of the risk stratification system.

### 3.4 SHAP analysis

The SHapley Additive exPlanations (SHAP) method was applied to the XGBoost model. Figure 3 presents a comprehensive SHAP summary plot, which integrates global feature importance (represented by the length of the overlaid bar charts) with the distribution of individual local impacts (represented by the beeswarm scatter points). Globally, Age, the Age-adjusted Charlson Comorbidity Index (ACCI), and lower limb mobility (Lowermob) were the top predictors of incident disability. Depression also ranked highly, surpassing specific mechanical limitations like upper limb mobility (Uppermob) and stair-climbing difficulty (Climsa). At the individual level, higher values (yellow/orange points) for ACCI, Lowermob, Depression, and Uppermob consistently mapped to positive SHAP values, indicating an elevated disability risk. Conversely, lower values (purple points) for these features clustered negatively, acting protectively. Notably, while difficulty walking 100 meters (Walk100 m) ranked lowest in overall importance due to its low baseline prevalence, its presence generated extreme positive SHAP values (>0.25). This highlights severe walking limitations as a critical independent warning signal.

**Figure 3:**
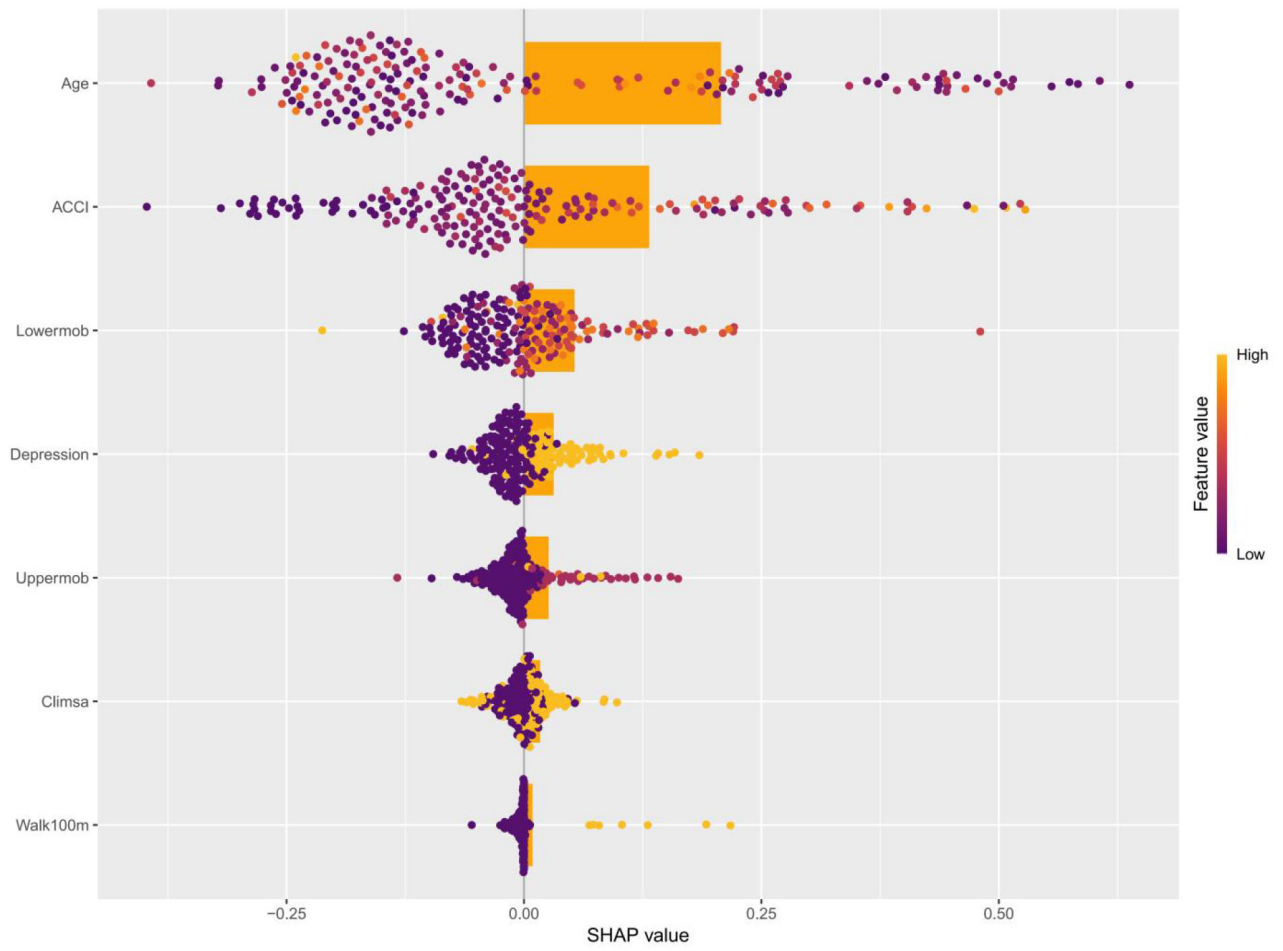
SHAP analysis for the XGBoost model. SHAP values represent each feature's contribution to the individual prediction (0 = baseline; positive values indicate higher disability risk and negative values lower risk), with color denoting the feature's raw value from low (purple) to high (yellow).

### 3.5 Risk stratification and DCA analysis

To facilitate personalized clinical interventions, the continuous predicted probabilities generated by the XGBoost model were translated into an actionable three-tier risk stratification system. The cut-offs of 0.3317 and 0.6523 yielded the highest chi-square statistic (χ^2^ = 2849.68). Based on optimal values, the cohort was stratified into Low-risk (probability < 0.3317), Moderate-risk (0.3317 ≤ probability < 0.6523), and High-risk (probability ≥ 0.6523) categories. The complete grid search results, including all threshold combinations and their corresponding chi-square statistics, are provided in Supplementary Appendix 6. The robustness of this stratification system was rigorously evaluated in the independent external validation cohort, ensuring its reliability under real-world, imbalanced class distributions. As illustrated in Table 3, the actual incidence of disability increased significantly across the predefined risk tiers in the external cohort. A Cochran-Armitage trend test confirmed a highly significant dose-response relationship between the assigned risk tiers and the observed disability outcomes (*P*_trend_ < 0.001). Baseline characteristics of the three risk groups in the external validation cohort are summarized in Supplementary Appendix 7.

**Table 3:** Incidence of disability and trend analysis across the defined risk strata in the external testing set.

Risk level	Total *N* per tier	Observed events	Observed rate	*P*_trend_
Low risk	411	8	0.019	< 0.001
Middle risk	1,470	100	0.068	
High risk	185	97	0.524	

To evaluate the clinical applicability and net benefit of the optimal XGBoost model, Decision Curve Analysis (DCA) was conducted (Figure 4). The DCA curve demonstrates that applying the XGBoost model to guide early disability interventions yields a higher net clinical benefit than either the “treat-all” or “treat-none” strategies across an exceptionally wide range of threshold probabilities (from 0% to approximately 70%). Based on the established risk stratification system, both of these designated thresholds fall firmly within the positive net benefit range of the DCA curve. Even at the highly conservative high-risk threshold of 65.23%, the XGBoost model maintains a positive clinical net benefit.

**Figure 4:**
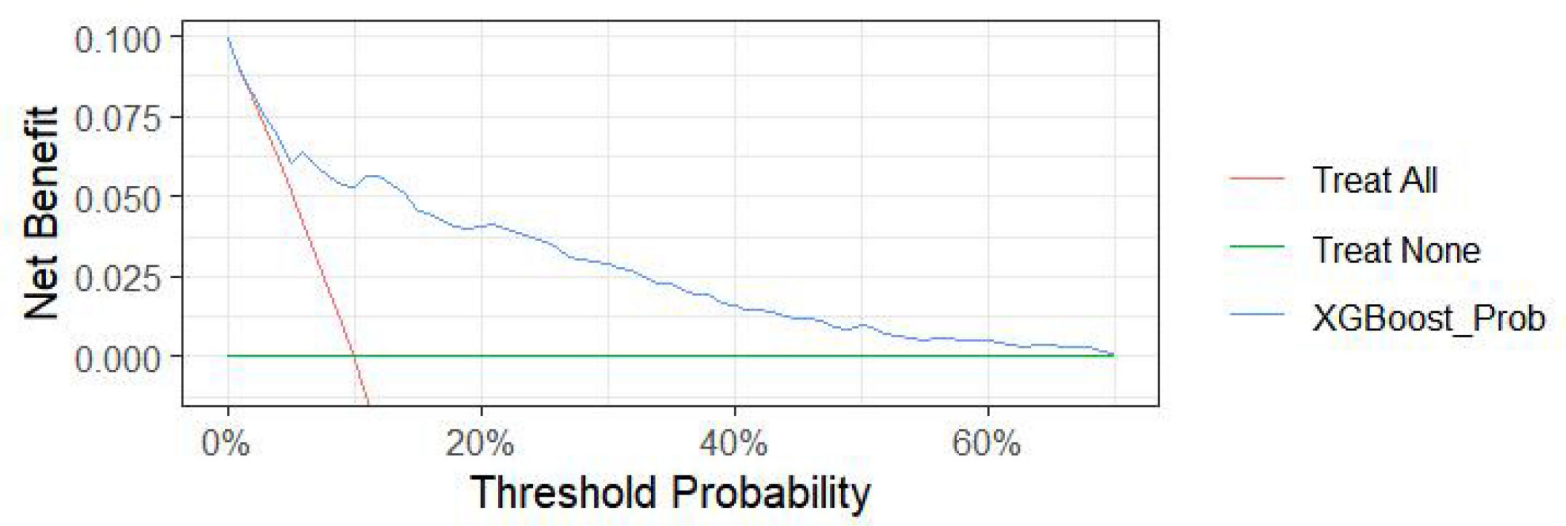
The Decision Curve Analysis (DCA) curve.

## 4 Discussion

In this study, we systematically developed and compared five machine learning models based on longitudinal data from CHARLS to predict the risk of disability among older adults in China, and validated these models in an independent external community cohort. The results showed that the XGBoost model outperformed other algorithms in both internal and external validation. Using the SHAP method, we further revealed the direction and strength of contributions from key predictors at both the individual and population levels. Age, the age-adjusted Charlson Comorbidity Index (ACCI), and lower limb mobility impairment are the three most important variables for predicting the risk of disability. Additionally, we established a three-tier risk stratification system based on model-predicted probabilities and confirmed its gradient validity in the external validation. This study provides a promising and practical tool for the precise identification and tiered intervention of disability risk among older adults.

Previous studies have attempted to develop predictive models for functional decline in older adults. Zhang et al. developed a physical performance-based model for predicting Activities of Daily Living (ADL) disability using CHARLS data; the model achieved a maximum AUC of 0.732, but no external validation was conducted (10). Their model relied heavily on physical performance measures [handgrip strength, gait speed, and Short Physical Performance Battery (SPPB)] while incorporating fewer sociodemographic, comorbidity, and psychological variables. Additionally, the issue of sample imbalance was not addressed. Han and Wang reported a higher AUC of 0.803 using the same CHARLS data, but their model required extensive laboratory biomarkers (e.g., white blood cell count) that are not routinely available in community-based primary care settings, limiting practical applicability (14). Besides, similar to Zhang et al., Han and Wang's study lacked independent external validation, raising potential concerns about overfitting. Xiang et al. (13) used XGBoost with SHAP to predict disability in older patients with hypertension, reporting AUCs of 0.759, 0.728, and 0.694 for 4-, 6-, and 10-year predictions, respectively; however, their model was developed specifically for hypertensive patients and may not generalize to the broader older adult population. Wu et al. applied random forests with SHAP to evaluate individual and community characteristics, but did not perform systematic model comparison or risk stratification (15).

In contrast to these studies, our work has several distinguishing features. First, our model was developed using LASSO for feature selection and systematically compared five machine learning algorithms, with XGBoost achieving an external validation AUC of 0.782—comparable to or better than the performance reported in similar studies. We employed the SMOTE algorithm to effectively mitigate this bias, thereby improving the model's sensitivity in identifying individuals with disabilities. Second, we prioritized easily accessible predictors (age, ACCI, mobility limitations, depressive symptoms) to enhance clinical utility in primary care settings. Third, and most importantly, we provided independent external validation in a geographically distinct cohort from Zhejiang Province—a step that was notably absent in the studies discussed above. In our external validation, the sensitivity of random forest dropped sharply to 0.286, while XGBoost maintained a sensitivity of 0.712, reflecting the latter's robustness to class imbalance in real-world data. Fourth, unlike most existing studies that only produce binary probability predictions, we translated the continuous predicted probabilities into a validated three-tier risk stratification system that may be used to guide practice.

Notably, the external validation cohort exhibited some differences from the development cohort across multiple baseline characteristics, including lower prevalences of depression, arthritis, and functional limitations. We interpret these differences as reflecting genuine population heterogeneity. First, the external validation sample represents a population from economically developed regions with better healthcare access, higher health literacy, and more proactive health management, whereas CHARLS covers 28 provinces including many less-developed inland and rural areas. Second, the nearly 10-year gap between the two data collections means that the external cohort has benefited from substantial advancements in community-based care for older adults, chronic disease management, and health promotion in China. This population heterogeneity also provides a test of the model's real-world robustness and cross-regional applicability.

SHAP analysis revealed that age, age-adjusted Charlson Comorbidity Index (ACCI), and lower limb mobility impairment are the most important predictors of disability. Our findings on these core predictors are consistent with a recent scoping review of frailty onset prediction models, which identified age, comorbidity burden, and physical function as the most frequently cited predictors across the literature (32). Chronological age, as an unmodifiable risk factor, has been considered as the strongest risk factor for aging-related death and disease (16, 33–35). According to research, centenarians' health declines far more slowly before the age of 85 than that of non-centenarians; multimorbidity, disability, and cognitive impairment start four to nine years later. However, in centenarians, the accumulation of chronic illnesses, disabilities, and cognitive deterioration accelerates dramatically beyond age 85 (36). These findings suggest that the impact of age on disability is not a simple linear relationship, but rather may be mediated through aging-related processes and reduced physiological functional reserves.

Age-adjusted Charlson comorbidity index (ACCI)—as a multimorbidity assessment tool, it is closely related to various adverse outcomes, such as fractures, malnutrition, and even death (37–39). The primary mechanism by which comorbidity burden contribute to disability may be attributed to the reduction of the body's physiological reserve. The potential ability of cells, organs, or systems to adapt to variations in physiological demand is known as physiological reserve (40). It's crucial for managing health stressors and figuring out how much disruption a person can handle (41). Individuals' physiological reserves may decline gradually due to natural aging and underlying diseases (42). Moreover, multiple chronic diseases not only damage the physiological system but also pose significant challenges for patients in long-term self-management. When self-efficacy declines, it may be associated with poor recovery and disease progression, which in turn may contribute to the progression toward disability (43).

Limited lower limb mobility is also one of the most important predictors. It is worth noting that in the SHAP plot, once the difficulty of walking 100 meters exists, it will trigger a very high positive SHAP risk value. Functional impairment, as an early indicator of disability risk, appears to have an immediate impact once it occurs (44). This trend suggests that daily independence is directly threatened by reduced basic physical functioning. An abrupt reduction in lower-body mobility can significantly reduce an older adult's level of independence (45). From a biological perspective, these limitations are intrinsically linked to underlying musculoskeletal and neurological decline, which is typically a direct result of the accumulation of chronic diseases or the natural aging process (46, 47).

Furthermore, a notable finding in the SHAP analysis is the prominent role of psychological status. Depressive symptoms ranked higher in overall importance than specific mechanical limitations, such as upper limb mobility and stair-climbing difficulty. Older adults with depressive tendencies tend to adopt maladaptive coping strategies when faced with health-related stressors (48). Such negative attitudes may cause a significant decline in personal motivation and physical activity levels, which in turn may result in clinical physical dysfunction and disability (49). In a study of older adults in a Portuguese community, Gaspar et al. reported that mental health (such as depressive symptoms) and cognitive reserve are important predictors of reversible and potentially reversible cognitive frailty (50). Although their study focused on cognitive frailty rather than disability, the emphasis on psychological factors is consistent with the results of our SHAP analysis, suggesting that depressive symptoms play a central role in accelerating functional decline in older adults.

Beyond elucidating the underlying predictive logic through SHAP analysis, another major clinical contribution of this study is overcoming the limitations of conventional binary predictions. The majority of recent research can only make binary probability predictions about whether a disability exists or not, which fails to meet the need for personalized interventions. To address this, our study developed a refined three-tier risk stratification system categorizing older adults into low, moderate, and high-risk levels. This system has been validated by our external cohort, demonstrating a significant gradient trend toward incident disability and yielding substantial clinical net benefits, as evidenced by the Decision Curve Analysis (DCA).

Finally, the distribution of risk categories within our external validation cohort warrants further discussion. Our samples were drawn from community-dwelling older adults in Zhejiang Province. Unlike hospital-based clinical cohorts, community populations generally exhibit better health, with the vast majority falling into low- or moderate-risk categories. Therefore, identifying a relatively small proportion of individuals (approximately 9%) as high-risk aligns with real-world epidemiological characteristics. Although the high-risk cohort is numerically small, the actual incidence of disability within this group was exceptionally high (52.4%). This underscores the model's satisfactory precision in pinpointing the most vulnerable minority, ensuring that intensive and scarce medical resources are concentrated strictly on those with the greatest clinical need. Conversely, the large population at moderate risk also constitutes a key early-warning group; for this group, scalable and low-cost strategies—such as enhanced routine monitoring and targeted lifestyle interventions—are most appropriate. By matching distinct risk strata with graded public health interventions, this three-tier system may help maximize overall healthcare efficiency.

While our model demonstrates acceptable predictive performance and mechanistic interpretability, several limitations should be acknowledged. First, the CHARLS data used for model development are self-reported, and some variables (such as depressive symptoms) may be subject to recall bias or subjective bias. Second, the baseline characteristics of the external validation set differ significantly from those of the development set. While this to some extent validates the model's generalization ability, it also suggests that the model's calibration may vary across different populations. Third, baseline disability status and some variables were determined by retrospective recall, which may introduce recall bias. However, some predictor variables were derived from objective healthcare records, mitigating the concern of recall bias. Also, as the baseline was reconstructed using historical records and recall rather than prospective enrollment, this validation does not constitute a perfect prospective temporal validation. Fourth, this study did not include other potentially relevant variables, such as social support structures. Fifth, although we used multiple imputation to handle missing data and the missing proportions were generally low, we cannot completely rule out the possibility that the missingness pattern may have introduced some degree of bias. Sixth, the Cochran-Armitage trend test assumes a strictly linear trend in proportions across risk tiers. While this assumption is widely used to validate dose-response relationships in epidemiological studies, we acknowledge that the true relationship between predicted probabilities and observed disability may not be perfectly linear. Finally, although we have established a three-tier risk stratification system, its clinical effectiveness—such as its ability to effectively guide tiered interventions—has not yet been validated in prospective practice.

Overall, this study successfully achieved its primary objective of constructing an interpretable, externally validated risk stratification framework for incident disability. Several avenues for future research emerge from these findings for gerontological medicine. Stratified prediction models enable healthcare providers to shift toward proactive, precision medicine-based management, thereby allowing them to efficiently allocate scarce geriatric healthcare resources based on stratified risk. Moving forward, future research should focus on prospective, multi-center longitudinal studies to capture the dynamic trajectories of key predictors over time, incorporating repeated measures of functional status and comorbidity burden to potentially improve predictive accuracy beyond static baseline assessments. Furthermore, randomized controlled trials are essential to rigorously evaluate the real-world clinical efficacy, cost-effectiveness, and long-term outcomes of implementing this tiered intervention strategy within community health systems. Ultimately, integrating such predictive algorithms into routine electronic health records may help pave the way for automated, real-time disability risk surveillance in the aging population.

## 5 Conclusion

In summary, this study successfully developed, interpreted, and externally validated a generally robust machine learning framework for predicting the risk of disability in older adults. The optimized XGBoost model demonstrated reasonable predictive performance and cross-cohort transferability, particularly maintaining the screening sensitivity required in real-world clinical settings. The integration of SHAP analysis unlocked the “black box” of the machine learning model, underscoring the critical predictive roles of age, comorbidity burden, lower limb mobility, and depressive symptoms. Most importantly, translating continuous probability scores into a validated three-tier risk stratification system provides a potentially practical and cost-effective tool for geriatric health management. This framework may help optimize the allocation of healthcare resources and empowers healthcare providers and policymakers to implement early, personalized, and tiered interventions, thereby contributing to promoting healthy aging and potentially delaying the onset of disability.

## Data Availability

The raw data supporting the conclusions of this article will be made available by the authors, without undue reservation.
