# Supplementary material for: Development and external validation of a machine learning model for three-tier disability risk stratification in older adults
Source: Front Public Health. 2026 Sep 18;14:1904998. doi: 10.3389/fpubh.2026.1904998 (PMC13630684; doi:10.3389/fpubh.2026.1904998)
Supplement: Supplementary file 1 [file Table_1.docx]

**Development and External Validation of a Machine Learning Model for Three-Tier Disability Risk Stratification in Older Adults**

| **Number** | **Content** | **Page** |
| --- | --- | --- |
| Appendix 1 | TRIPOD Checklist: Prediction Model Development and Validation | 2-3 |
| Appendix 2 | The detailed codebook for 46 variables. | 4-8 |
| Appendix 3 | The work flow of this study | 9 |
| Appendix 4 | Proportion of missing values in baseline variables | 10 |
| Appendix 5 | The Pearson correlation heatmap | 11 |
| Appendix 6 | Grid search results for risk stratification cutoff points | 12 |
| Appendix 7 | Comparison of baseline features for 7 key predictive variables stratified by risk level in the external validation set | 13 |

# Appendix 1 TRIPOD Checklist: Prediction Model Development and Validation

| **Section/Topic** | **Item** |  | **Checklist Item** | **Location** |
| --- | --- | --- | --- | --- |
| **Title and abstract** | | | | |
| Title | 1 | D;V | Identify the study as developing and/or validating a multivariable prediction model, the target population, and the outcome to be predicted. | Title |
| Abstract | 2 | D;V | Provide a summary of objectives, study design, setting, participants, sample size, predictors, outcome, statistical analysis, results, and conclusions. | Abstract |
| **Introduction** | | | | |
| Background and objectives | 3a | D;V | Explain the medical context (including whether diagnostic or prognostic) and rationale for developing or validating the multivariable prediction model, including references to existing models. | Introduction, Paragraph 1-2 |
|  | 3b | D;V | Specify the objectives, including whether the study describes the development or validation of the model or both. | Introduction Paragraph 3 |
| **Methods** | | | | |
| Source of data | 4a | D;V | Describe the study design or source of data (e.g., randomized trial, cohort, or registry data), separately for the development and validation data sets, if applicable. | Methods,  Section 2.1 |
|  | 4b | D;V | Specify the key study dates, including start of accrual; end of accrual; and, if applicable, end of follow-up. | Methods,  Section 2.2.1-2.2.2 |
| Participants | 5a | D;V | Specify key elements of the study setting (e.g., primary care, secondary care, general population) including number and location of centres. | Methods,  Section 2.2.1-2.2.2 |
|  | 5b | D;V | Describe eligibility criteria for participants. | Methods,  Section 2.2.1-2.2.2 |
|  | 5c | D;V | Give details of treatments received, if relevant. | N/A |
| Outcome | 6a | D;V | Clearly define the outcome that is predicted by the prediction model, including how and when assessed. | Methods,  Section 2.3 |
|  | 6b | D;V | Report any actions to blind assessment of the outcome to be predicted. | N/A |
| Predictors | 7a | D;V | Clearly define all predictors used in developing or validating the multivariable prediction model, including how and when they were measured. | Methods,  Section 2.4 |
|  | 7b | D;V | Report any actions to blind assessment of predictors for the outcome and other predictors. | N/A |
| Sample size | 8 | D;V | Explain how the study size was arrived at. | Methods,  Section 2.2.3 |
| Missing data | 9 | D;V | Describe how missing data were handled (e.g., complete-case analysis, single imputation, multiple imputation) with details of any imputation method. | Methods,  Section 2.5 |
| Statistical analysis methods | 10a | D | Describe how predictors were handled in the analyses. | Methods,  Section 2.5 |
|  | 10b | D | Specify type of model, all model-building procedures (including any predictor selection), and method for internal validation. | Methods,  Section 2.5 |
|  | 10c | V | For validation, describe how the predictions were calculated. | Methods,  Section 2.5 |
|  | 10d | D;V | Specify all measures used to assess model performance and, if relevant, to compare multiple models. | Methods,  Section 2.5 |
|  | 10e | V | Describe any model updating (e.g., recalibration) arising from the validation, if done. | N/A |
| Risk groups | 11 | D;V | Provide details on how risk groups were created, if done. | Methods,  Section 2.5.3 |
| Development vs. validation | 12 | V | For validation, identify any differences from the development data in setting, eligibility criteria, outcome, and predictors. | Table 1 |
| **Results** | | | | |
| Participants | 13a | D;V | Describe the flow of participants through the study, including the number of participants with and without the outcome and, if applicable, a summary of the follow-up time. A diagram may be helpful. | Appendix 3 |
|  | 13b | D;V | Describe the characteristics of the participants (basic demographics, clinical features, available predictors), including the number of participants with missing data for predictors and outcome. | Table 1 |
|  | 13c | V | For validation, show a comparison with the development data of the distribution of important variables (demographics, predictors and outcome). | Table 1 |
| Model development | 14a | D | Specify the number of participants and outcome events in each analysis. | Table 1 |
|  | 14b | D | If done, report the unadjusted association between each candidate predictor and outcome. | Appendix 5 |
| Model specification | 15a | D | Present the full prediction model to allow predictions for individuals (i.e., all regression coefficients, and model intercept or baseline survival at a given time point). | Results,  Section 3.4 |
|  | 15b | D | Explain how to the use the prediction model. | Results,  Section 3.5 |
| Model performance | 16 | D;V | Report performance measures (with CIs) for the prediction model. | Table 2 |
| Model-updating | 17 | V | If done, report the results from any model updating (i.e., model specification, model performance). | N/A |
| **Discussion** | | | | |
| Limitations | 18 | D;V | Discuss any limitations of the study (such as nonrepresentative sample, few events per predictor, missing data). | Discussion,  Paragraph 11 |
| Interpretation | 19a | V | For validation, discuss the results with reference to performance in the development data, and any other validation data. | Discussion |
|  | 19b | D;V | Give an overall interpretation of the results, considering objectives, limitations, results from similar studies, and other relevant evidence. | Discussion |
| Implications | 20 | D;V | Discuss the potential clinical use of the model and implications for future research. | Discussion,  Paragraph 12 |
| **Other information** | | | | |
| Supplementary information | 21 | D;V | Provide information about the availability of supplementary resources, such as study protocol, Web calculator, and data sets. | Yes |
| Funding | 22 | D;V | Give the source of funding and the role of the funders for the present study. | Yes |

# Appendix 2 The detailed codebook for 46 variables.

| **Categories** | **Variables** | **Code meanings** |
| --- | --- | --- |
| Sociodemographic variables (*n* = 6) | Gender | 0= Male  1= Female |
|  | Age | Years |
|  | Marriage | 1= married (including living and not living with spouse)  2= widowed  3= divorced  4= never married |
|  | Education | 1= Less than lower secondary education, e.g. "No Formal Education (Illiterate)", "Did Not Finish Primary School but can Read", "Sishu (Private Tutoring)", "Elementary School" or "Middle School".  2= Upper secondary & vocational training, e.g. "High School" or "Vocational School"  3= Tertiary education, e.g."Two/three-year College", "College Grad" or "Post-graduate degree" |
|  | Retirement | 0= engage in agricultural work, non-agricultural employed work, non-agricultural self-employed work, or non-agricultural unpaid family business work, or unemployed or never worked per their labor force status.  1= have retired per their labor force status. |
|  | Accident | 0= having never received medical treatment as the result of a traffic accident or another kind of major accidental injury.  1= having ever received medical treatment as the result of a traffic accident or another kind of major accidental injury. |
| Lifestyle variables (*n* = 4) | Smoke | 0= never smoke or quit smoking  1= smoke now |
|  | Drink | 0= never have any alcoholic beverage in the last 12 months  1= have had an alcoholic beverage in the last 12 months |
|  | Drinking-frequency | 0= none during the last year  1= less than once a month  2= 1 to 3 days a month  3= 1 to 4 days a week  4= equal to or more than 5 days a week |
|  | Social participation | 0 = did not participate in any of the social activities in the past month.  1= participated in at least one of the social activities, including: interacted with friends, played Ma-jong, played chess, played cards, or went to community club, went to a sport, social, or other kind of club, took part in a community-related organization, done voluntary or charity work or attended an educational or training course. |
| Family and economic variables  *N* = 4 | Family number | The number of individuals in the house |
|  | Live with child | 0= do not live with their children  1= live with their children |
|  | Lvnear | 0= the respondent and spouse have living children but none of these children co-reside with nor live in the same city or county as the respondent and spouse.  1= any child co-resides with the respondent and spouse or any non-co-residing child lives in the same city or county as the respondent and  spouse. |
|  | Economic support received | 0= never receive financial support from their children  1=have received financial support from their children |
| Comorbidities and hospitalization (*n* = 12) | Hypertension | 0= don’t have hypertension  1= have hypertension |
|  | Stroke | 0= never have a stroke  1= ever have a stroke |
|  | Diabetes | 0= never have diabetes  1= have diabetes or high blood sugar |
|  | Lung disease | 0= don’t having chronic lung disease such as chronic bronchitis or emphysema (excluding tumors, or cancer)  1= having chronic lung disease such as chronic bronchitis or emphysema (excluding tumors, or cancer) |
|  | Cancer | 0=never have any cancer  1=having cancer or a malignant tumor (excluding minor skin cancers) |
|  | Psychiatric illnesses | 0= never have any emotional, nervous, or psychiatric problems  1= have any emotional, nervous, or psychiatric problems |
|  | Heart disease | 0= never have heart attack, coronary heart disease, angina, congestive heart failure, or other heart problems  1= have heart attack, coronary heart disease, angina, congestive heart failure, or other heart problems |
|  | Digestive disease | 0= never have any stomach or other digestive disease (except for tumor or cancer)  1= have any stomach or other digestive disease (except for tumor or cancer) |
|  | Arthritis | 0= never have arthritis or rheumatism  1= have arthritis or rheumatism |
|  | Memory-related diseases | 0= never have memory-related diseases  1= have memory-related diseases |
|  | ACCI | Age-adjusted Charlson Comorbidity Index  ACCI scores were calculated by the method previously reported by Charlson, in which comorbid conditions are weighted and scored, with additional points added for age (Table 1). |
|  | Hospitaliztion | The number of times that the respondent has received inpatient care in the  past year. |
| Physical examinations (*n* = 8) | BMI | Body Mass Index (kg/m2) |
|  | BMI-level | The BMI category according to the WHO recommendation for the Asian and South Asian population.  1=underweight (less than 18.5)  2=normal weight (18.5 to less than 23)  3=overweight (23 to less than 25)  4=obesity (25 or greater) |
|  | Waist | waist measurement in centimeters |
|  | Abdomina-obesity | 0=waist less than 90 for male/85 for female  1=waist more than 90 for male/85 for female |
|  | Sbsdone | SBSTAN indicates the amount of time the respondent held the side-by-side test position in seconds to two decimal places. The respondent was instructed to stand with feet together, side-by-side, for 10 seconds. The respondent was allowed to use their arms, bend their knees, or move their body to maintain balance. Only respondents who participated in the semi-tandem test and were not able to maintain balance for 10 seconds in the semi-tandem test performed the side-by-side test.  0= the respondent maintained their balance below 10 seconds  1= the respondent's time more than 10 seconds (It also defaults to respondents who have completed either the semi-tandem standing test or the full-tandem test.) |
|  | Semidone | SEMIDONE indicates whether the respondent maintained their balance for the full 10 seconds during the semi-tandem standing test.  0= the respondent maintained their balance below 10 seconds  1= the respondent's time more than 10 seconds. |
|  | Fulldone | FULLDONE indicates whether the respondent maintained their balance for the full 30/60 seconds during the full-tandem standing test. Respondents aged 70 and over were only expected to hold full-tandem for 30 seconds, but their full time was recorded if past 30 seconds, while those under 70 years old were expected to hold 60 seconds, if able.Only respondents who maintained the semi-tandem position for the full 10 seconds performed the full-tandem test.  1= the respondent maintained their balance for the full time  0= the respondent's time fell below the expected time for their age group. |
|  | Banlance | BALANCE indicates the assigned highest balance level of the respondent.  1= did not complete the semi-tandem nor side-by-side test to the maximum time.  2 = did not complete the semi-tandem test to the maximum time, but did complete the side-by-side test to the maximum time.  3=completed the semi-tandem test to the maximum time, but did not complete the expected full-tandem test time.  4=completed the semi-tandem test and full-tandem test to the maximum time. |
| Health status and functional limitations (*n* = 12) | Walk100m | 0= did not have any problems with walking 100m  1= have some difficulty with walking 100m or could not finish |
|  | Stoopa | 0= did not have any problems with stooping kneeling, or crouching  1= have some difficulty with stooping kneeling, or crouching or could not finish |
|  | Climsa | 0= did not have any problems with climbing several flights of stairs without resting  1= have some difficulty with climbing several flights of stairs without resting or could not finish |
|  | Chaira | 0= did not have any problems with getting up from a chair after sitting for long periods  1= have some difficulty with getting up from a chair after sitting for long periods or could not finish |
|  | Lowermob | LOWERMOB is a 4-item summary of any difficulty with lower-body mobility activities. The lowerbody mobility activities included are walking 100m, climbing several flights of stairs, getting up from a chair, and stooping or kneeling or crouching, ranging from 0 to 4. |
|  | Armsa | 0= did not have any problems with reaching arms above shoulder level  1= have some difficulty with reaching arms above shoulder level or could not finish |
|  | Lifta | 0= did not have any problems with lifting or carrying weights over 10 jins  1= have some difficulty with lifting or carrying weights over 10 jins or could not finish |
|  | Dimea | 0= did not have any problems with picking up a coin from the table  1= have some difficulty with picking up a coin from the table or could not finish |
|  | Uppermob | UPPERMOB is a 3-item summary of any difficulty with upper-body mobility activities. The upperbody mobility activities included are reaching arms above shoulder level, lifting or carrying weights over 10 jins, and picking up a coin from the table, ranging from 0 to 3. |
|  | Mobilsev | MOBILSEV is a 7-item summary of any difficulty with mobility activities. The mobility activities included are walking 100m, climbing several flights of stairs, getting up from a chair, stooping or kneeling or crouching, extending arms up, lifting 10 jin, and picking up a small coin,ranging from 0 to 7. |
|  | Orientation | 0=the respondent is able to recall the day of the month, the month ,the year and the day of the week correctly.  1=the respondent is unable to recall correctly any items, including the day of the month, the month ,the year and the day of the week. |
|  | Depression | According to Radloff’s CESD-10,The total score is calculated by finding the sum of 10 items. Any score equal to or above 10 is considered depressed.  0= total score less than 10  1= total score equals to or above 10 |

Table 2 Aged-adjusted Charlson Comorbidity Index

| **Weight** | **Comorbid condition** |
| --- | --- |
| 1 | Myocardial infarction  Congestive heart failure  Peripheral vascular disease  Cerebral vascular disease  Dementia  Chronic obstructive pulmonary disease  Connective tissue disease  Ulcer disease  Mild liver disease  Diabetes |
| 2 | Hemiplegia  Moderate/severe renal disease  Diabetes with end-organ damage  Any tumor  Leukemia  Lymphoma |
| 3 | Moderate/severe liver disease |
| 6 | Metastatic solid tumor  AIDS( acquired immune deficiency syndrome) |
| 1 | For each decade over age 40 y |

The corresponding points are added if a patient has any of the aforementioned conditions. All relevant points must be tallied together because a single patient may have more than one comorbidity. Additionally, points are added according to the patient's age; for those above 40, one point is added for each ten years that their age increases.

# Appendix 3 The work flow of this study.

# Appendix 4 Proportion of missing values in baseline variables

# Appendix 5 The Pearson correlation heatmap

# Appendix 6 Grid search results for risk stratification cutoff points

| **Q1** | **Q2** | **T1** | **T2** | **Chi square** | ***P*** | **Rate_low** | **Rate_medium** | **Rate_high** |
| --- | --- | --- | --- | --- | --- | --- | --- | --- |
| 0.46 | 0.6 | 0.3317 | 0.6523 | 2849.68 | ＜0.001 | 7.7 % | 49.7 % | 98.8 % |
| 0.48 | 0.6 | 0.3759 | 0.6523 | 2847.58 | ＜0.001 | 8.7 % | 53.0 % | 98.8 % |
| 0.44 | 0.6 | 0.2917 | 0.6523 | 2839.39 | ＜0.001 | 7.0 % | 46.5 % | 98.8 % |
| 0.48 | 0.62 | 0.3759 | 0.7452 | 2829.24 | ＜0.001 | 8.7 % | 57.4 % | 99.5 % |
| 0.5 | 0.6 | 0.3929 | 0.6523 | 2827.56 | ＜0.001 | 9.9 % | 55.9 % | 98.8 % |
| 0.46 | 0.62 | 0.3317 | 0.7452 | 2824.84 | ＜0.001 | 7.7 % | 54.0 % | 99.5 % |
| 0.42 | 0.6 | 0.2593 | 0.6523 | 2816.24 | ＜0.001 | 6.5 % | 43.2 % | 98.8 % |
| 0.5 | 0.62 | 0.3929 | 0.7452 | 2814.39 | ＜0.001 | 9.9 % | 60.5 % | 99.5 % |
| 0.44 | 0.62 | 0.2917 | 0.7452 | 2807.49 | ＜0.001 | 7.0 % | 50.6 % | 99.5 % |
| 0.52 | 0.6 | 0.4254 | 0.6523 | 2807.14 | ＜0.001 | 11.1 % | 59.7 % | 98.8 % |
| 0.52 | 0.62 | 0.4254 | 0.7452 | 2799.89 | ＜0.001 | 11.1 % | 64.5 % | 99.5 % |
| 0.4 | 0.6 | 0.2314 | 0.6523 | 2798.57 | ＜0.001 | 6.0 % | 40.5 % | 98.8 % |
| 0.48 | 0.64 | 0.3759 | 0.8231 | 2793.67 | ＜0.001 | 8.7 % | 62.2 % | 100.0 % |
| 0.5 | 0.64 | 0.3929 | 0.8231 | 2785.92 | ＜0.001 | 9.9 % | 65.5 % | 100.0 % |
| 0.46 | 0.64 | 0.3317 | 0.8231 | 2780.67 | ＜0.001 | 7.7 % | 58.7 % | 100.0 % |
| 0.38 | 0.6 | 0.2092 | 0.6523 | 2779.47 | ＜0.001 | 5.7 % | 38.1 % | 98.8 % |
| 0.52 | 0.64 | 0.4254 | 0.8231 | 2779.36 | ＜0.001 | 11.1 % | 69.6 % | 100.0 % |
| 0.54 | 0.6 | 0.4676 | 0.6523 | 2779.22 | ＜0.001 | 12.3 % | 64.3 % | 98.8 % |
| 0.54 | 0.62 | 0.4676 | 0.7452 | 2777.73 | ＜0.001 | 12.3 % | 69.2 % | 99.5 % |
| 0.42 | 0.62 | 0.2593 | 0.7452 | 2776.34 | ＜0.001 | 6.5 % | 47.2 % | 99.5 % |

Note：Q1 and Q2 denote the percentiles of the predicted probability distribution used to generate candidate thresholds. T1 and T2 are the corresponding probability cut-offs for distinguishing low-risk from medium-risk (T1) and medium-risk from high-risk (T2), respectively. Rate_low, Rate_medium, and Rate_high represent the observed disability incidence (%) within each risk stratum under the given threshold pair. *P* values are derived from Chi-square tests comparing the disability rates across the three risk groups for each candidate cut-off combination.

Due to the large number of combinations generated during the grid search process, this table only lists the top 20 parameter combinations, ranked in descending order by the Chi-square statistic. As shown in the table, setting the cutoff points at the 46th and 60th percentiles of the predicted probability (corresponding to 33.17% and 65.23%, respectively) yielded the global maximum Chi-square value (2849.68).

# Appendix 7 Comparison of baseline features for 7 key predictive variables stratified by risk level in the external validation set

| **Variable** | **Level** | **Low risk group**  **(*N* = 411)** | **Moderate risk group**  **(*N* = 1,470)** | **High risk group**  **(*N* = 185)** | ***P*** |
| --- | --- | --- | --- | --- | --- |
| Age | / | 76.00  [74.00, 79.50] | 71.00  [68.00, 76.00] | 80.00  [71.00, 90.0] | ＜0.001 |
| ACCI | / | 1.00  [0.00, 1.00] | 4.00  [3.00, 5.00] | 6.00  [3.00, 7.00] | ＜0.001 |
| Walking difficulty | No | 410 (99.8) | 1409 (95.9) | 75 (40.5) | ＜0.001 |
|  | Yes | 1 ( 0.2) | 61 ( 4.1) | 110 ( 59.5) |  |
| Stair climbing difficulty | No | 410 (99.8) | 1414 (96.2) | 105 (56.8) | ＜0.001 |
|  | Yes | 1 ( 0.2) | 56 ( 3.8) | 80 ( 43.2) |  |
| Number of difficulties in lower limb movement | 0 | 388 ( 94.4) | 1277 ( 86.9) | 78 ( 42.2) | ＜0.001 |
|  | ≥1 | 23 ( 5.6) | 193 ( 13.1) | 107 ( 57.8) |  |
| Number of difficulties in upper limb movement | 0 | 392 ( 95.4) | 1364 ( 92.8) | 137 ( 74.1) | ＜0.001 |
|  | ≥1 | 19 ( 4.6) | 106 ( 7.2) | 48 ( 25.9) |  |
| Depression | No | 411 (100.0) | 1444 (98.2) | 150 (81.1) | ＜0.001 |
|  | Yes | 0 (0.0) | 26 (1.8) | 35 (18.9) |  |
| Disability | No | 403 ( 98.1) | 1370 ( 93.2) | 88 ( 47.6) | ＜0.001 |
|  | Yes | 8 (1.9) | 100 (6.8) | 97 ( 52.4) |  |
